# Supplementary material for: Splice-Junction-Based Mapping of Alternative Isoforms in the Human Proteome
Source: Cell Rep. Author manuscript; Available in PMC 2020 Jan 15. (PMC6961840; doi:10.1016/j.celrep.2019.11.026)
Supplement: 2 [file NIHMS1546469-supplement-2.zip › DF1/PXD010154/Spleen/Spleen_9_DUS1L_PQPAAPGTPGGFSEVMGSALR.pdf]

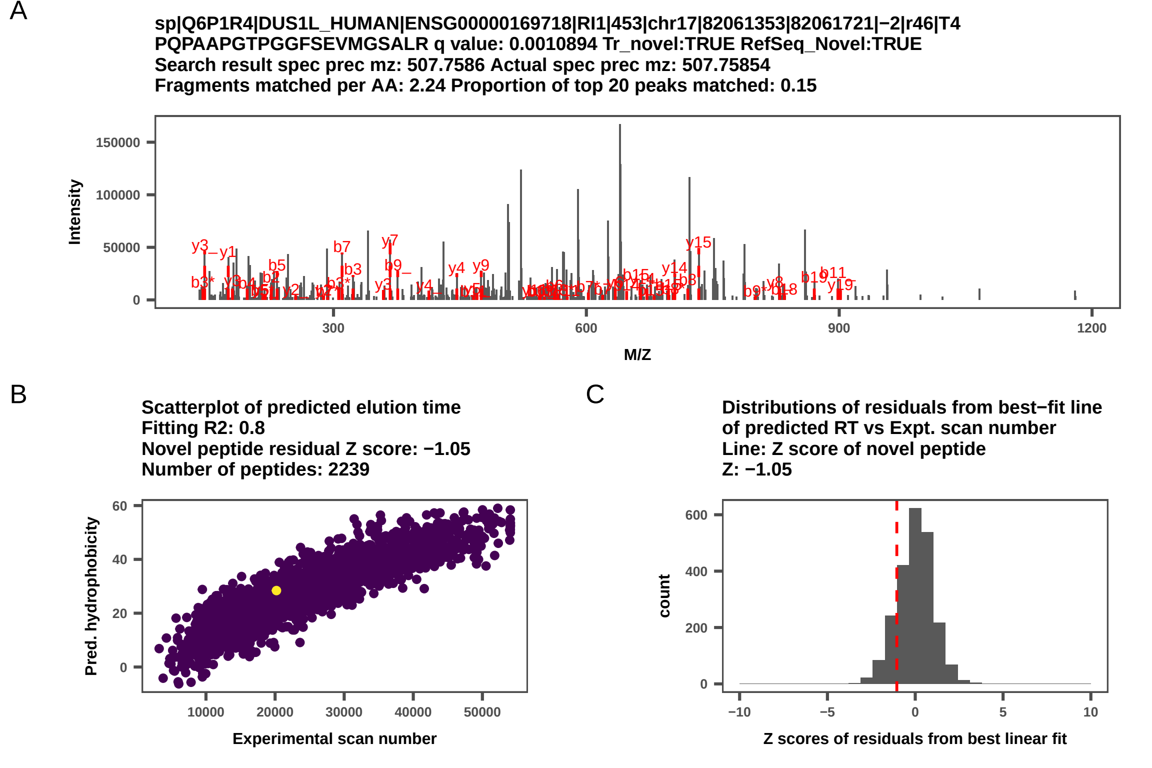
A
sp|Q6P1R4|DUS1L_HUMAN|ENSG00000169718|RI1|453|chr17|82061353|82061721|−2|r46|T4
PQPAAPGTPGGFSEVMGSALR q value: 0.0010894 Tr_novel:TRUE RefSeq_Novel:TRUE
Search result spec prec mz: 507.7586 Actual spec prec mz: 507.75854
Fragments matched per AA: 2.24 Proportion of top 20 peaks matched: 0.15
Intensity
150000
100000
50000
0
300
600
900
1200
M/Z
y3
y1
b3*
b5
b7
b3
y7
b9
y15
y14
y4
y9
b19
b11
y19
y8
B
Scatterplot of predicted elution time
Fitting R2: 0.8
Novel peptide residual Z score: −1.05
Number of peptides: 2239
Pred. hydrophobicity
60
40
20
0
10000
20000
30000
40000
50000
Experimental scan number
C
Distributions of residuals from best−fit line
of predicted RT vs Expt. scan number
Line: Z score of novel peptide
Z: −1.05
count
600
400
200
0
−10
−5
0
5
10
Z scores of residuals from best linear fit
